# N-Channel MOSFET Reliability Issue Induced by Visible/Near-Infrared Photons in Image Sensors †

**DOI:** 10.3390/s23239586

**Published:** 2023-12-03

**Authors:** Chun-Hsien Liu, Sheng-Di Lin

**Affiliations:** Institute of Electronics, National Yang Ming Chiao Tung University, Hsinchu 300, Taiwan; sdlin@nycu.edu.tw

**Keywords:** light-induced reliability, transistor degradation, parasitic BJT action, CMOS image sensor, single-photon avalanche diode (SPAD)

## Abstract

Image sensors such as single-photon avalanched diode (SPAD) arrays typically adopt in-pixel quenching and readout circuits, and the under-illumination first-stage readout circuits often employs high-threshold input/output (I/O) or thick-oxide metal-oxide-semiconductor field-effect transistors (MOSFETs). We have observed reliability issues with high-threshold n-channel MOSFETs when they are exposed to strong visible light. The specific stress conditions have been applied to observe the drain current (I_d_) variations as a function of gate voltage. The experimental results indicate that photo-induced hot electrons generate interface trap states, leading to I_d_ degradation including increased off-state current (I_off_) and decreased on-state current (I_on_). The increased I_off_ further activates parasitic bipolar junction transistors (BJT). This reliability issue can be avoided by forming an inversion layer in the channel under appropriate bias conditions or by reducing the incident photon energy.

## 1. Introduction

In imaging applications, to enhance image quality and resolution, the pursuit of larger arrays and smaller pixel pitch is a recognized trend. In-pixel circuit design is considered one of many suitable solutions to achieve this goal. In-pixel circuit with n-type metal-oxide-semiconductor field-effect transistors (NMOSFETs), such as in the four-transistor active pixel sensor (4T-APS) [1], are widely employed. In the front-end circuits of single-photon avalanche diodes (SPADs), similar approaches effectively reduce the spacing between pixels so the fill factor (FF) can be increased [2]. To further enhance the FF of imaging sensor array with in-pixel circuits, the on-chip micro-lenses have been introduced to achieve a nearly 100% FF [3,4]. However, the previous work showed that the micro-lenses could increase the energy density of incident light by at least 50%, which raised concerns about the reliability of devices and circuits when exposed to high-intensity light sources and/or strong ambient illumination [5].

In the design of readout circuits for SPAD arrays, high-voltage-tolerant input/output (I/O) NMOSFETs are typically used to reach high excess bias voltage [6,7]. In a few chips with similar design, we have observed that, after long exposure to a white-light light-emitting diode (LED), the digital signal processor (DSP) within the SPAD chip without bandpass filter was unable to extract signals from the front-end readout circuit and resulted in a system malfunction [8]. In general, the circuit area in pixel region is optically shielded by the metal layer in CMOS technology to avoid optical damage, but the damage still occurred in these circuits with an optical shield. Surprisingly, with the same illumination condition, the packaged chip seemed much more vulnerable to optical damage compared to those that were unpackaged. We reckon that the circuits may be exposed to the light with a large/diverse incident angle and the multiple reflections in the package. While temporary functionality could somehow be restored by increasing the input signal amplitude, eventually, the instability problem of the front-end circuits for signal readout could not be resolved. This indicates that the photo-induced damage arises from a long term or even permanent mechanism. For the applications involving SPAD sensors, such as RGB image sensors, X-ray imaging, and radiometric temperature detection, the operational wavelengths are not limited to the near-infrared spectrum; they can extend into the visible or even shorter wavelengths. In these scenarios, the use of a long-pass filter in front of the SPAD sensor is not applicable. Therefore, in this present work, we investigate the impact of the light-induced reliability issues on NMOSFET under intense light exposure. The dependence of degradation on the device structure, the stress condition, the illumination method, and the incident photon energy are studied. A qualitative model to explain the NMOSFET degradation as well as the possible ways to mitigate this reliability issue are also presented.

## 2. Experimental Methods and Results

**Figure 1:**
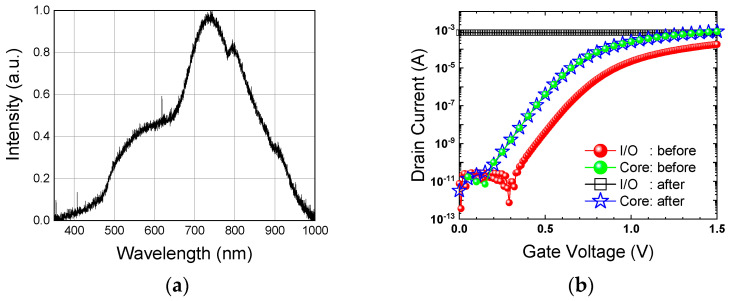
(**a**) Tungsten lamp spectrum measured by Ocean USB4000-UV-VIS-ES. (**b**) I_d_-V_g_ curves measured in darkness after and before stress sweeping V_g_ from 0 V to 1.5 V under CW illumination for I/O and core NMOSFETs.

The devices were fabricated using the HEJIAN 0.11 μm high-voltage process. Two kinds of NMOSFETs were examined, including I/O and core devices. They have the same gate width of 4.0 μm and gate length of 0.45 μm. All devices are not optically shielded by the metal layer. The differences between the I/O and core devices are (1) the I/O devices have a thicker oxide layer than the core devices, and (2) there is no lightly doped drain (LDD) structure under the spacer at the source/drain for the I/O MOSFETs. In order to quantify the issue of light-induced reliability, we first measure the drain current (I_d_) as a function of the gate voltage (V_g_) in the dark after a period of stress. The stress tests involve different bias voltages and illumination modes. The stress condition for NMOSFET is set in common with a source amplifier, similar to that under which a SPAD readout circuit is operated. During the stress, we choose two electrical bias methods: DC bias (DC mode) and square-wave operation (AC mode). On the other hand, the optical stress also involves two testing modes: continuous wave (CW) and pulsed illumination. For the following experiments, a tungsten lamp is used as the light source for illumination. The light intensity is 3.6 klux measured by a lux meter (CHY-331) and the normalized intensity spectrum is shown in Figure 1a.

Figure 1b shows the I_d_-V_g_ curves of two devices before and after the stress of 2000 times I_d_-V_g_ sweeps under CW illumination. V_g_ sweeps from 0.0 V to 1.5 V with a step of 10 mV and 50 mV for the I/O and core NMOSFETs, and the time for one sweep is about 3 s. For the I/O NMOSFET, its drain current clearly increases to about 1 mA after stress and exhibits no switching characteristics at all. In strong contrast, the core NMOSFET maintains the same I_d_-V_g_ curve after the same stress condition. It is obvious that I_d_ degradation is only observed with the I/O NMOSFET. In order to elucidate the degradation mechanism, we have applied the following three stress conditions based on the aforementioned parameters:

1. V_g_ in DC mode under CW illumination.
2. V_g_ in DC mode under pulsed illumination.
3. V_g_ in AC mode under pulsed illumination.

**Figure 2:**
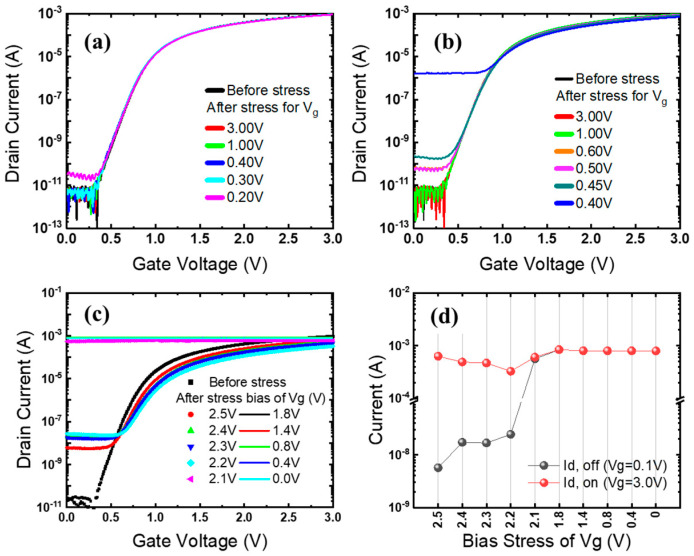
I_d_-V_g_ curves in darkness before and after stress of (**a**) DC mode, (**b**) AC mode under CW illumination, and (**c**) AC mode under pulsed illumination. (**d**) On- and off-state current (I_on_) versus V_g_ in stress condition.

In order to clearly illustrate the differences before and after applying stress, the following experiments present the results by measuring the I_d_-V_g_ curves with V_g_ sweeping from 0.0 V to 3.0 V in 10 mV increments at V_d_ = 1.5 V. Figure 2a illustrates the I_d_-V_g_ curves of I/O NMOSFETs after the stress. The device is stressed for 100 s using stress condition 1 simply with fixed V_g_ (DC mode). The V_g_ is fixed at 3.0 V at the beginning, then decreased by a step of 0.1 V. The degradation appears in the I_d_-V_g_ measurement for V_g_ coming to 0.2 V as the off-state current (I_off_) increases from ~20 pA to ~200 pA. In Figure 2b, with stress condition 2, the degradation occurs earlier at a larger V_g_ of 0.6 V. By changing the illumination from CW into pulsed mode, the NMOSFET degradation occurs at a higher V_g_, and a much worse I_off_ is obtained.

For stress condition 3, when the gate voltage is operated in AC mode under pulsed illumination mode, the degradation of I_d_ is the worst, as shown in Figure 2c. The pulsed-mode illumination is a periodic on- and off-state with a frequency of about 1 kHz and duty cycle of 50% obtainable by turning on/off the lump power controller. The degradation starts as early as Vg = 2.5 V and is noticeably different from the previous two stress conditions. After the stress, Ioff gradually increases, while the on-state current (Io_n_) initially decreases, then increases, as shown in Figure 2d. The serious degradation of the drain current causes the NMOSFETs to lose its switching characteristics. Even worse, the leakage current leads to a very high static power consumption on the chip. After a week of idle time, we found that the degraded NMOSFETs are still unable to exhibit their normal I_d_-V_g_ curves, indicating that this degradation is not recoverable.

## 3. Analysis and Discussion

**Figure 3:**
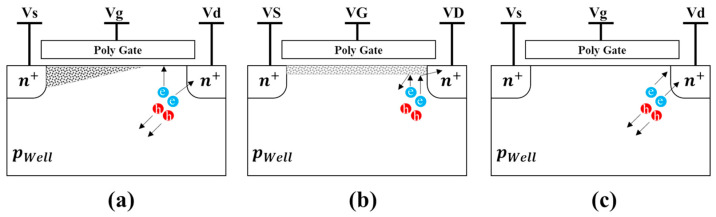
Hot electrons damage mechanism in (**a**) saturation, (**b**) linear, and (**c**) cut-off states under illumination.

The degradation of I_d_ may be attributed to an increase in surface trap density. Figure 3 illustrates the possible mechanism to be detailed in the following. When exposed to light, electron–hole pairs are generated, and higher-energy photons can potentially produce high-energy electrons. Although the drain electric field in NMOSFET is not sufficient to induce the hot carrier injection (HCI) effect, the high-energy electron-hole pairs are accelerated by the drain electric field, resulting in carriers with enough energy to cause damage to the chemical bonds at the Si/SiO_2_ interface. The weak Si-H bonding could be broken by hot electrons, leading to the emergence of interface traps [9,10]. This degradation mechanism appears to be valid only when the V_g_ is significantly smaller than the drain voltage (V_d_). This is because when V_g_ is much smaller than V_d_, the NMOSFET enters the saturation region, and pinch-off occurs on the drain side. This exposes the interface oxide layer that is originally surrounded by the inverted n-channel, making it vulnerable to hot electron damage, as shown in Figure 3a. When NMOSFET is biased in the linear region, the strong electric field disappears, so the hot electrons stop damaging the SiO_2_/Si interface as shown in Figure 3b. For V_g_ = 0 V, the hot electrons would not be attracted toward the gate, and therefore, no device degradation occurs, as shown in Figure 3c. Additionally, the high density of hot-electron-induced interface traps affects the overlap region of the drain and the gate, which could cause band bending and enhance I_off_ due to the trap-assisted tunneling current [11].

To understand why the pulsed illumination induces the degradation more easily compared to the CW one, we reckon that the continuous flow of the photo-current may play a protective role. The I_d_ degradation in NMOSFET is due to the photon-induced hot electrons, but at the same time, they could also provide a form of protection. Under CW illumination, a stable photo-current flows from the drain to the substrate. This photo-current could protect the Si/SiO_2_ interface from the direct impact of the hot electrons. The continuous flow of photo-carriers generated by CW illumination could increase the probability of scattering and lead to a decrease in hot-electron energy, so the damage on the interface could be reduced. Consequently, the degradation phenomenon under CW mode is not very pronounced. As the illumination mode is switched from CW to pulsed mode, the continuous photo-current needs to be re-established at each on/off transition of the illumination, so the protection of the photo-current flow is significantly weakened. This is why a clear increase in the threshold for I_d_ degradation is observed when the illumination is switched from CW to pulsed mode.

**Figure 4:**
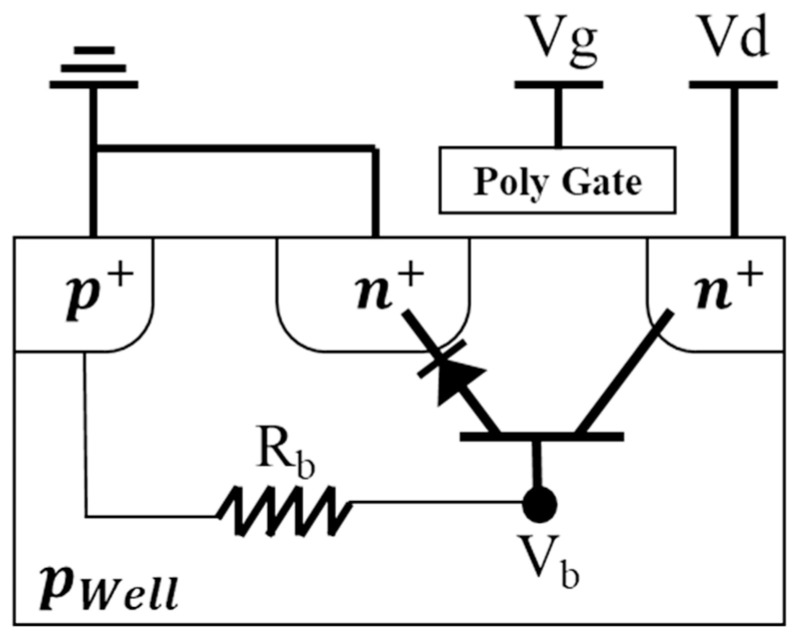
Parasitic bipolar junction transistor (BJT) in NMOSFET.

As I_off_ gradually increases, the parasitic resistance of the substrate, influenced by the body effect, provides a voltage at the substrate. Within the NMOSFET structure, there is a parasitic bipolar junction transistor (BJT) located between the source, the body, and the drain, as sketched in Figure 4. When the parasitic diode between the substrate and source becomes forward-biased due to the substrate voltage, the parasitic BJT is activated, allowing a current to flow from the drain to the source. This new current path is not influenced by the gate voltage and accounts for the loss of switching characteristics, as seen in Figure 2c.

To generate hot electrons near the channel, the incident photon energy has to be high enough. To confirm that photon energy is one of the primary factors for causing the observed interface damage, the 700 nm and 900 nm long-pass filters (LPFs) are placed at the light source output to selectively filter incoming photon energy. Because the HEJIAN devices were all measured and damaged in the previous experiments, the following experiment used new devices as a replacement. The new devices of I/O NMOSFETs were fabricated using TSMC’s 0.18 μm bipolar-CMOS-DMOS (BCD) process. The respective sizes in gate length and gate width are 4 μm and 0.6 μm.

**Figure 5:**
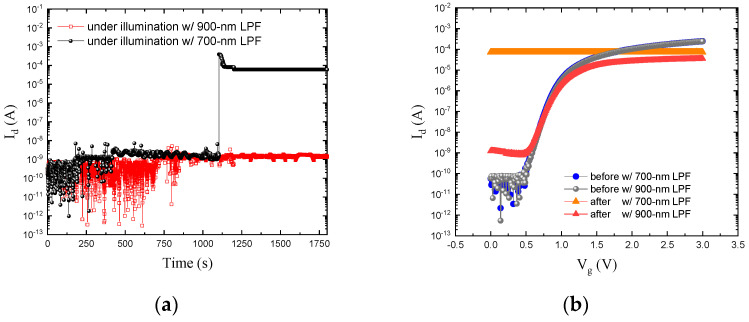
(**a**) Drain current versus time under stress of DC mode under pulsed illumination and (**b**) I_d_-V_g_ curves in darkness before and after the stress with and without 700 nm and 900 nm long pass filter.

Figure 5a,b show the stress effect with the two LPFs using stress condition 1 for 30 min at V_g_ = 0.3 V and V_d_ = 1.5 V. The drain current during the stress is monitored, and it is clear that only the device in the experiment with 700 nm LPF exhibits an abrupt jump of I_d_ at about 1150 s as shown in Figure 5a. The significant difference in I_d_-V_g_ before and after the stress can also be seen in Figure 5b. The NMOSFET using the 700 nm LPF exhibited severe I_d_ degradation, while the ones using the 900 nm filter only occurred slight damage, with I_d_ increasing to ~nA after the stress. This evidences that photon energy is a key factor influencing the I_d_ degradation mechanism.

Since an LPF determines the maximum energy photons that can pass through, when hot electrons are generated, their own energy combined with the acceleration provided by the vertical electric field within the structure meets the condition for causing interface damage, similar to the hot-electron injection effect. Therefore, shorter-wavelength photons can generate higher-energy hot electrons to induce the interface damage. In addition, the wavelength of photons decides the absorption depth. Note that the photons with shorter wavelengths have a shallower absorption depth, and a large number of electrons generated by these photons are close to the surface of the device, making them more likely to damage the surface oxide.

**Figure 6:**
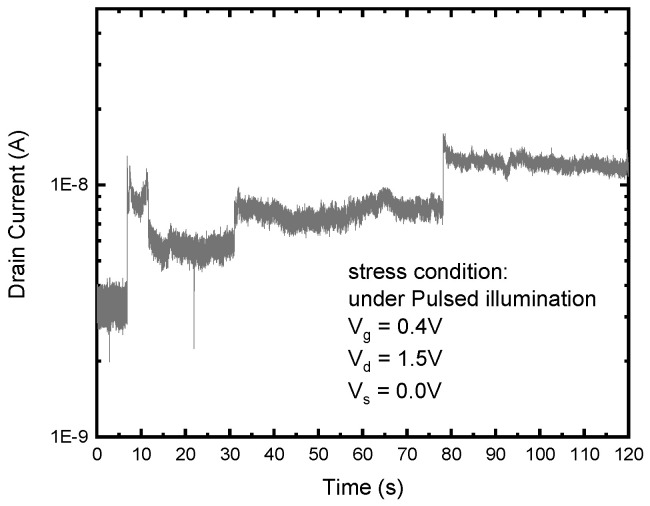
Monitored drain current as a function of the stress time under stress condition 2 at Vg = 0.4 V.

Hot carriers can hit the Si/SiO_2_ interface, causing the breakage of Si-H bonds and an increase in surface defects. Interestingly, hydrogen ions, restricted by the bias voltage of V_g_, are pushed back towards the interface. Therefore, in the early stages of damage, it is possible to observe the phenomenon of Si-H bonds breaking and re-forming. One of the cases spotted by us is shown in Figure 6. The monitored I_d_ values under stress condition 2 at V_g_ = 0.4 V from 0 to 120 s are plotted. The RTS-like up-and-down I_d_ has been observed in Figure 6, probably indicating a recovered interface damage. Among several measurements, we also found that these RTS-like I_d_ behaviors become more seldom as V_g_ is lowered. This can be understood because at lower V_g_ values, it is more difficult for the hydrogen ions to return to the interface for recombination. Consequently, we may observe recoverability in the early stages of degradation, but it eventually results in permanent damage.

## 4. Conclusions

We have presented the photon-induced reliability problems in NMOSFET using in SPADs or CMOS image sensors. Under the illumination of the light source, photons of high energy can be fully absorbed into surface and generate hot electrons that are accelerated by the vertical electric field and impact the Si/SiO_2_ interface. The damage induces high-density interface traps contributing to the increased I_off_. The enlarged I_off_ then activates parasitic BJTs and creates a new current path from the source to the drain of the NMOSFET. The worst case occurs at sweeping V_g_ and pulsed illumination and leads to a failure of NMOSFET on/off characteristics. In addition, the short-wavelength photons generate a large number of electrons on the surface due to their shallower absorption depth, significantly increasing the probability of damaging the interface. A 900 nm long-pass filter above the device can largely reduce the damage as the incident photon energy is lower and the surface absorption is also lower. Our work shows that by choosing low-V_th_ transistors for circuit designs or using long-pass filters, the reliability issues associated with I_d_ degradation in NMOSFET can be effectively mitigated.

## Data Availability

The data that support the findings of this study are available from the corresponding author upon reasonable request.
